# Micro-Alloying Effects on Microstructure and Weldability of High-Strength Low-Alloy Steel: A Review

**DOI:** 10.3390/ma18051036

**Published:** 2025-02-26

**Authors:** Jian Chen, Zhongran Shi, Xiaobing Luo, Feng Chai, Tao Pan, Guanghong Feng, Caifu Yang

**Affiliations:** 1 Key Laboratory of Nonferrous Metal Materials Science and Engineering (Ministry of Education), School of Materials Science and Engineering, Central South University, Changsha 410083, China; 2 Institute of Structural Steels, Central Iron and Steel Research Institute, Beijing 100081, China

**Keywords:** micro-alloying elements, coarse-grained heat-affected zone, microstructure, weldability, high-strength low-alloy steel

## Abstract

High-strength low-alloy (HSLA) steels have garnered significant attention owing to their widespread applications across various industries, with weldability being a particularly critical aspect. However, the impact toughness of the coarse-grained heat-affected zone (CGHAZ) remains a notable challenge under high-heat-input welding conditions. Despite existing research acknowledging the beneficial effects of micro-alloying elements on steel properties, there are still numerous uncertainties and controversies regarding the specific influence of these elements on the microstructure and impact toughness of the CGHAZ under specific welding conditions. To address this issue, this study presents a comprehensive review of the impact of common micro-alloying elements on the microstructure and toughness of the CGHAZ during high-heat-input welding. The results indicate that elements such as cerium, magnesium, titanium, vanadium, nitrogen, and boron significantly improve the toughness of the CGHAZ by promoting intragranular nucleation of acicular ferrite and inhibiting the coarsening of austenite grains. In contrast, the addition of elements such as aluminum and niobium adversely affect the toughness of the CGHAZ. These findings offer crucial theoretical guidance and experimental evidence for further optimizing the welding performance of HSLA steels and enhancing the impact toughness of the CGHAZ.

## 1. Introduction

High-strength low-alloy (HSLA) steels have garnered significant attention in the field of engineering and industrial, including additive manufacturing [1,2], aerospace [3], shipbuilding [4], and oil and gas pipeline transportation [5], due to their exceptional blend of superior strength [6,7], ductility [8,9], and weldability [10]. In the context of manufacturing complex structures and large-scale components, welding that can be categorized as solid-state welding [11] and fusion welding [12] plays a pivotal role in joining parts and ensuring the integrity of the final product [13,14]. For steel with increased thickness, high-heat-input welding techniques are often employed to boost production efficiency and optimize resource utilization [15,16]. However, this process introduces unique challenges, particularly in the heat-affected zone (HAZ), where rapid heating and cooling cycles result in the formation of non-equilibrium microstructures [17,18]. The HAZ, which encompasses distinct regions including the fusion zone, coarse-grained heat-affected zone (CGHAZ), fine-grained or recrystallization heat-affected zone (FGHAZ), and intercritical heat-affected zone (ICHAZ), undergoes significant microstructural transformations during welding [19,20]. Among these, the CGHAZ, located adjacent to the weld seam, is particularly susceptible to detrimental effects such as grain coarsening, an increase in martensite-austenite (M/A) islands, and the development of brittle microstructures. These changes undermine the microstructural integrity and properties of the CGHAZ, rendering it the weakest zone in the steel structure and compromising its overall service reliability [21,22,23,24]. Given the critical role of the HAZ in determining the performance of HSLA steels, enhancing its properties has emerged as a crucial challenge.

To mitigate the adverse effects of high-heat-input welding on the HAZ, one promising approach involves the incorporation of micro-alloying elements into HSLA steel with lower carbon equivalents, in conjunction with advanced technologies like oxide metallurgy [25,26,27]. Over the past decades, oxide metallurgy technology has emerged as a pivotal approach for enhancing the low-temperature impact toughness of the HAZ. This technological advancement is attributed to the concerted efforts and explorations of numerous researchers. Various studies have scrutinized the evolution of oxide metallurgy technology from diverse perspectives. Notably, Mu et al. [28] conducted comprehensive analyses of the intrinsic relationship between inclusions and microstructures, unveiling the transformation mechanisms of these microstructures. Shao et al. [29] focused on elucidating the formation mechanism of intragranular acicular ferrite (IAF), systematically reviewing the significant influences of austenitizing conditions, continuous cooling rates, and isothermal quenching parameters on the IAF transformation. Ma et al. [30] further investigated which inclusions can effectively function as nucleation sites for IAF. Additionally, Liang et al. [31] outlined the background of oxide metallurgy technology and emphasized the potential application of rare earth metals in optimizing HAZ microstructures. Recently, the influence of micro-alloying elements on IAF nucleation and HAZ microstructures under oxide metallurgy conditions has garnered significant research attention, with the aim of further improving the low-temperature impact toughness of the HAZ. These micro-alloying additions not only compensate for the strength loss associated with lower carbon concentrations but also contribute to microstructural refinement, precipitation hardening, and solid solution strengthening [32]. Furthermore, the non-metallic inclusions formed during welding at high-heat-input conditions can restrict austenitic grain growth and promote the formation of acicular ferrite (AF), thereby enhancing the toughness of the HAZ [33]. For example, Xu et al. [34] found that adding Mg to shipbuilding steel significantly inhibits the growth of prior austenite grains and accelerates the formation of fine, interwoven IAF and polygonal ferrite (PF), leading to enhanced HAZ toughness after welding at 400 kJ cm^−1^. Wang et al. [35] observed that co-deoxidation with Ti and Zr produces small, complex inclusions that act as effective nucleation sites for IAF, resulting in significantly improved HAZ toughness compared to C-Mn steel without Ti or Zr addition after welding at 100 kJ cm^−1^.

However, a comprehensive and systematic summary of the IAF nucleation mechanism and the synergistic effects of multiple alloying elements remains elusive. Therefore, this review, based on the latest research findings, delves into the specific impacts of micro-alloying elements, such as cerium (Ce), aluminum (Al), magnesium (Mg), niobium (Nb), boron (B), vanadium (V), nitrogen (N) and titanium (Ti), on the welding performance of the HAZ after welding. The objective is to provide valuable insights and references for research and applications in related fields.

## 2. Effect of Micro-Alloying Elements on CGHAZ

### 2.1. Ce

**Figure 1:**
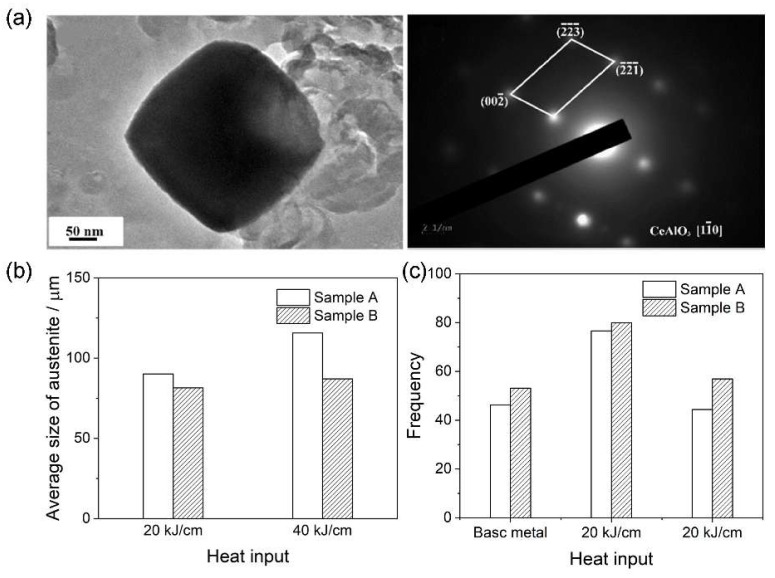
(**a**) Nano-sized Ce-containing inclusions in the CGHAZ. (**b**) Average sizes of the austenite grains in the CGHAZ at different heat inputs. (**c**) Frequency of high-angle grain boundaries with misorientation angles larger than 45° in samples A and B at different heat inputs [37].

Ce treatment is recognized as a promising approach to enhance the mechanical properties of HSLA steel [36,37,38]. The addition of Ce, which primarily exists as oxidation products, facilitates the formation of refined inclusions, resulting in enlargement of the equiaxed zone, refinement of the as-cast microstructures, and subsequent improvement in the welding performance of steels [37,39,40,41,42]. Recently, much research has focused on exploring the utilization of Ce in the microstructure and toughness of HAZ. Geng et al. [43] conducted a study to examine the influence of adding 0.0023% Ce on the mechanical properties of 700 MPa class Al-killed high-strength steel in the HAZ. By comparing the element mappings of typical inclusions in Ce-free and Ce-containing steels, they observed a transformation in the inclusions from a complex mix of CaS, Mg–Al–O, and Ti(C,N) in Ce-free steel to primarily Ce–Ca–O–S, CaS, and Ti(C,N) in Ce-containing steel. Furthermore, a notable shift in the size distribution of inclusions was observed upon the addition of Ce. Compared to the Ce-free steel, the proportion of inclusions smaller than 1 μm and larger than 2 μm increased significantly from 16.0% to 42.7% and 30.3% to 7.8%, respectively. Additionally, the number density of inclusions per square millimeter increased from 60 to 79.8, while their average size decreased from 1.71 μm to 1.13 μm. These modifications in inclusion characteristics, attributed to the refining effect of Ce, can pin on austenite grain boundaries and restrain austenite grain growth, leading to a substantial enhancement in the impact energy, yield strength, and tensile strength of the HAZ following welding operations with heat inputs of 25 and 50 kJ cm^−1^. Apart from the role of inclusions, Geng et al. [37] reported that the addition of Ce also affects the microstructure after heat treatment, second phases, and mechanical properties of steels. Compared to the Ce-free steel, Ce addition improved the toughness of the base metal and CGHAZ with an increase of 59% and 48% by increasing the crack propagation energy. During the welding thermal cycle, the Nb and Ti carbonitrides greatly affected the grain growth in the CGHAZ. Ce addition significantly refined the average size and number of (Nb,Ti)(C,N) carbonitrides. Additionally, the presence of micro-nano Ce-containing inclusions provides a stronger pinning pressure to inhibit austenite grain growth in the CGHA (Figure 1a). Thus, the average sizes of austenite grain in CGHAZ were decreased from 90.2 to 81.5 at a heat input of 20 kJ cm^−1^ and from 115.7 μm to 87.1 μm at 40 kJ/cm (Figure 1b) after adding Ce, respectively. Moreover, after Ce was added, the frequency of high-angle grain boundaries with misorientation angles larger than 45° in the base metal and CGHAZ was increased by 6.87%, 3.25% and 12.53% (Figure 1c), respectively. These results indicate that Ce addition also refined the austenite grains and increased the proportion of high-angle boundaries in the CGHAZ, thus increasing the impact absorb energy and decreasing the strength loss of the CGHAZ, essentially improving the welding performance of the 800 MPa HSLA steel.

### 2.2. Al

**Figure 2:**
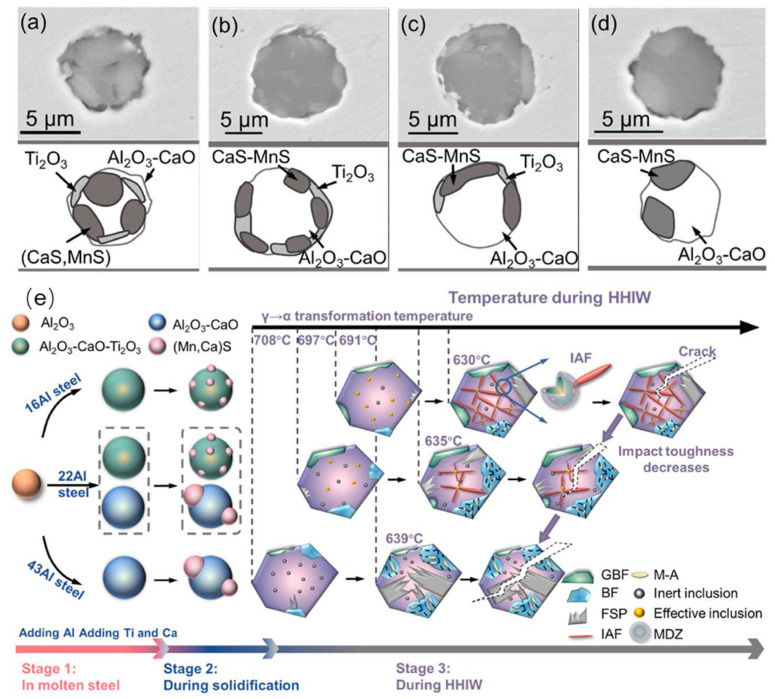
(**a**–**d**) Morphologies of the typical inclusions in the CGHAZs of (**a**,**b**) 16Al steel, (**c**) 22Al steel, and (**d**) 43Al steel. (**e**) Schematic illustrations of inclusion characteristics and ferrite transformation in CGHAZ after HHIW with 400 kJ/cm for the 16Al, 22Al, and 43Al steels [48].

In the production of HSLA steel plates, Al is intensively utilized as the primary deoxidizer in oxide metallurgy process due to its cost-effectiveness and strong thermodynamic affinity with oxygen [44]. The addition of aluminum (Al) exerts an indirect influence on the inclusion characteristics, microstructure, and impact toughness of the HAZ. Cui et al. [45] and Pan et al. [46] observed notable alterations in inclusion count and the remarkable increase in brittle microstructures after intruding Al (0.02 wt%) into Mg deoxidized steel plates. Specifically, the quantity of coarse TiN particles exceeding 0.5 μm expanded from 124 to 151 mm^3^, and the proportion of brittle microstructures escalated from 25% to 78%. This change was attributed to the heightened lattice mismatch between inclusions and *α*-Fe in the high-Al samples, which diminished their nucleation potential for AF, resulting in a decrease in AF proportion with increasing Al content. Moreover, due to the augmentation of dislocations between ferrite laths, the toughness of HAZ was markedly reduced after adding Al. The similar phenomorphans were observed by Xu et al. [47] and Li et al. [48] in their investigations into the impact of Al on the HAZ of Mg or Al–Ti–Ca deoxidized shipbuilding steel plates. They found that the increasing of Al narrowed the austenite stability region, elevated the transformation temperature from austenite to ferrite, and impeded the nucleation of IAF [48]. Furthermore, as Al content increased from 0.016% to 0.043 wt%, a notable decline was observed in both the quantity and inductive capacity of critical inclusion types pivotal for intragranular IAF nucleation, specifically Al_2_O_3_–CaO–Ti_2_O_3_–MnS(-CaS) inclusion particles encapsulated by MnS (Figure 2a–d). Additionally, the increased Al diminished austenitic stability region, leading to an elevation in the temperature required for the *γ* → *α* phase transformation during continuous cooling (Figure 2e). Both of these shifts resulted in a substantial decline in the impact toughness of the CGHAZ. Notably, with a welding heat input of 400 kJ cm^−1^, as Al content rose from 0.016 wt% to 0.022 wt% and 0.043 wt%, the impact toughness of the CGHAZ plummeted from 134 J to 78 J and 24 J at −40 °C, respectively. Based on these reports, under high-heat-input welding conditions, steel plates with lower Al content exhibited superior toughness within the HAZ.

Interestingly, Yu et al. [49] observed a contrasting phenomenon in their investigation regarding the influence of Al on the HAZ of commercial X70 steel plate. As the Al content increased from 0.023% to 0.070%, there was a notable decrease in the volume percentage of massive M–A constituents, from 0.96% to 0.176%, and a decrease in M–A constituents, from 6.12% to 3.43%. As a result of the decrease in the proportion and refinement of M–A constituents, the average Charpy absorbed energy exhibited a significant increase, from 17 J to 250 J, as the Al content rose from 0.023% to 0.070% under a high-heat-input of 200 kJ cm^−1^.

The differing conclusions observed can potentially be attributed to variations in steel plate types and compositions, along with welding processes and conditions. Firstly, different steel plate systems exhibit distinct sensitivities to Al. Furthermore, the composite deoxidizers formed by Al in conjunction with other deoxidizers, such as Mg-Ti-Ca, significantly alter the types and morphologies of inclusions within the steel, thereby influencing its welding performance. Secondly, factors such as the selection of welding processes, welding parameter settings, and welding environment all play crucial roles in determining the effect of Al in HAZ. For example, in welding processes involving high energy densities, Al may evaporate or burn off, thereby altering the composition and properties of the weld joint. Additionally, welding conditions such as heat input and cooling rate impact the diffusion and distribution of Al within the steel, subsequently affecting welding performance. Consequently, to thoroughly investigate the role and underlying mechanisms of Al and its concentration in high-strength low-alloy steel, further systematic research is required.

### 2.3. Mg

**Figure 3:**
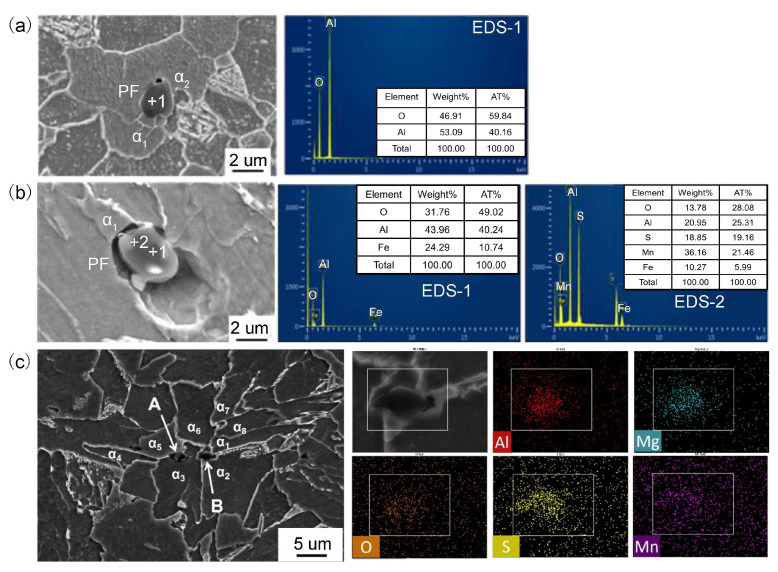
The relationship between inclusions and ferrite in the HAZ of No. 1 steel: (**a**) Al_2_O_3_; (**b**) Al_2_O_3_ + MnS; (**c**) mapping scanning results and the relationship between the Al–Mg–O + MnS and ferrite in the HAZ of Mg-treated steel at W2 heat input [19].

The addition of an optimal amount of Mg into Al-deoxidized low-carbon steel has been found to markedly enhance the toughness of CGHAZ [19,50,51]. Li et al. [50] reported that this enhancement after adding 0.0026% Mg primarily stems from the modification of inclusion and precipitate characteristics, including Mg-Al-O and (Nb,Ti)(C,N) + MgO compounds instead of Al_2_O_3_, as well as the Mg solid solution state. Additionally, the Al-Mg-O + MnS inclusions after Mg treatment can function as nucleation sites for AF, which not only contributes to the refinement of ferrite grains but also effectively inhibits the development of Widmanstätten ferrite and coarse-grained boundary ferrite [19], which may promote the advances of microstructure regulation techniques, and thus enhance the CGHAZ toughness. Based on the analysis of the relationship between typical inclusions and ferrite in the CGHAZ, two distinct types of ferrites are observed in the vicinity of Al_2_O_3_ inclusions: coarse PF (α1) and fine PF (α2) (Figure 3a). This indicates that following the submerged arc welding process, Al_2_O_3_ inclusions tend to reside within coarse PF rather than refined AF. As for Al_2_O_3_ + MnS composite inclusions, the presence of AF adjacent to the MnS component within these inclusions has been observed (Figure 3b). Regarding Al–Mg–O + MnS inclusions, numerous strip-like ferrites are present around inclusions A and B. Specifically, α3–α5 and α1–α2–α6 ferrites nucleate directly on the surfaces of these inclusions, respectively. Additionally, α4–α6 and α7–α8 are identified as sympathetic nucleation ferrites, forming on the surfaces of α3–α5 and α1–α2–α6 (Figure 3c), respectively. The co-inducing effect leads to the formation of interlaced and interlocked AF, thereby effectively preventing the development of coarse-grained boundary ferrite (GBF) and Widmannstätten ferrite (WF).

Under conditions of high-heat-input welding, the synergistic interaction between Mg and micro-alloying elements like Al and Ti can facilitate the formation of small composite inclusions and then induce the generation of IAF within the austenite matrix [52]. Thus, at a welding heat input of 150 kJ cm^−1^, the industrially tested 40 mm-thick shipbuilding steel plate exhibited an average impact absorbed energy of 198.9 J at a test temperature equivalent to laboratory conditions.

**Figure 4:**
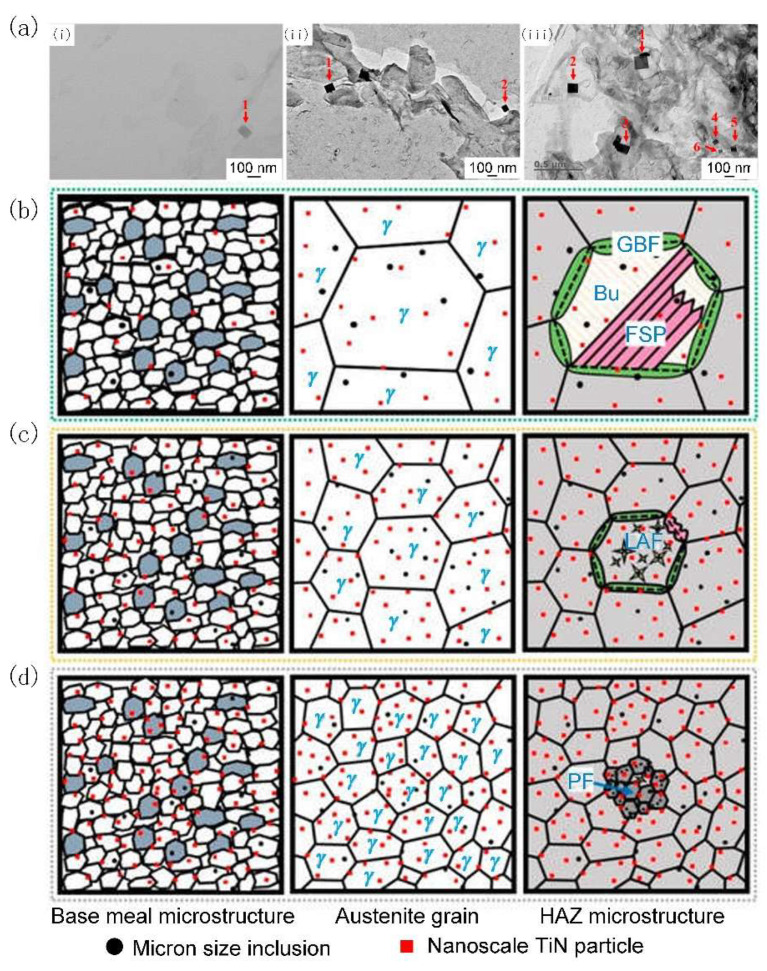
(**a**) Distribution of the nanoscale titanium nitride (TiN) particles in HAZs of (**i**) A, (**ii**) LM, and (**iii**) HM. (**b**–**d**) Schematic diagram of the mechanism for improving the HAZ toughness by inclusion control with Mg deoxidation: (**b**) A, (**c**) LM, and (**d**) HM. The black dots and the red square in the schematic diagram are micron-sized inclusions and nanoscale TiN particles [54].

Furthermore, Xu et al. [53,54] found that an increase in Mg content under high-heat-input welding conditions results not only in a rise in the number density but also a corresponding decrease in the average diameter of nanoscale TiN particles within the HAZ, contributing to the formation of IAF and PF. With increasing Mg, both the number density and the mean diameter of the nanoscale TiN particles increased (Figure 4a). This modification enhances the pinning effect, which serves to hinder the growth of austenite grains. Therefore, the size of austenite grains resulting from Al deoxidation (A) was larger than that from low-content Mg deoxidation (LM 0.0027 wt% of Mg) ((Figure 4a(i,ii) and Figure 4a,b). As for high-content Mg deoxidation (HM 0.0099 wt% of Mg), the presence of Mg significantly enhanced the formation of nanoscale TiN particles (Figure 4a(iii) and Figure 4d), resulting in a strong pinning effect for inhibiting the growth of austenite grains. During the phase transformation process, when the cooling rate was relatively slow, the formation of a fine PF microstructure in the HAZ of the HM sample was favored over the development of brittle microstructures (Figure 4d). After welding with a heat input of 400 kJ cm^−1^, the toughness of the HAZ of A, LM, and HM decreased by 88.59%, 25.66%, and 23.63%, respectively, compared with base metals. The improvements in the HAZ toughness of LM and HM are mainly attributed to the formation of IAF and PF. Therefore, an excellent HAZ toughness of steel plates after welding with a heat input of 400 kJ cm^−1^ could be obtained by control of the Mg content in steel to selectively promote the formation of IAF or inhibit the growth of austenite grains [54].

### 2.4. Nb

**Figure 5:**
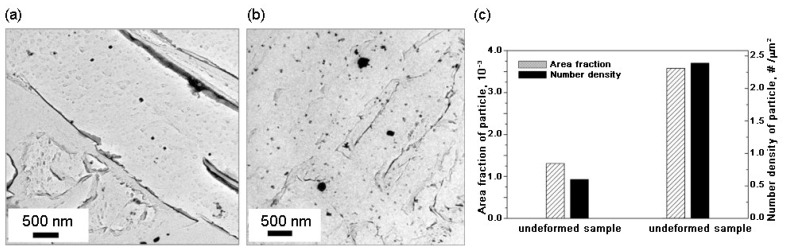
Comparison of particle distribution in the weld CGHAZ between a just thermal cycle without stress (undeformed process) and a thermomechanical cycle (undeformed process). (**a,b**) TEM micrographs in the CGHAZ: (**a**) undeformed case; (**b**) deformed case. (**c**) Area fraction and number density of particle distribution measured in undeformed and deformed samples [58].

To improve the mechanical properties of HSLA steel and achieve cost-effective production with superior quality, the incorporation of Nb as a micro-alloying element has gained widespread acceptance [55,56]. Nevertheless, this approach also has influences on the mechanical properties of the HAZ during high-heat-input welding processes. Moon et al. [57] investigated the influence of Nb addition on the HAZ in a Ti-containing steel weld and found that adding Nb triggers the coarsening of austenitic grains. This change is predominantly attributed to the diminished high-temperature stability of (Ti,Nb)(C,N) composite particles, which weakens their pinning effect and fosters the growth of austenitic grains. The author observed that following continuous thermomechanical cycling, the area fraction and number density of (Ti,Nb)(C,N) particles in CGHAZ with smaller average particle sizes exhibited a notable increase in comparison to those observed after thermal cycling only (Figure 5) [58]. This disparity arises from the intensified precipitation kinetics under deformation conditions [59], which stimulate the precipitation of finer particles, thereby partially mitigating the coarsening effects.

Apart from increasing the austenitic grains, Nb exerts a notable influence on ferrite transformation during high-heat-input welding process. Yang et al. [60] observed that Nb exhibits a tendency to segregate at the interfaces between micron-sized inclusions and the matrix, thereby exerting a detrimental influence on the nucleation potential of acicular ferrite. Furthermore, both Pan et al. [61] and Yang et al. [62] concurred that an elevated Nb content facilitates the formation of formation of low-temperature coarse intragranular bainite ferrite or coarse carbide bainite, which reduces the density of high-angled grain boundary (HAGB) in microstructures [63], thus significantly deteriorating the impact toughness of the HAZ. As the Nb in Mg-deoxidated shipbuilding steel plate increases to 0.016 wt%, the high-angled grain boundary density decreases from 1.3 to 0.5 μm^−1^ and the effective grain size increases from 10.4 to 17.6 μm after high-heat-input welding at 400 kJ cm^−1^, resulting in a decrease in toughness from 127 to 58 J at −40 °C [61].

### 2.5. B

**Figure 6:**
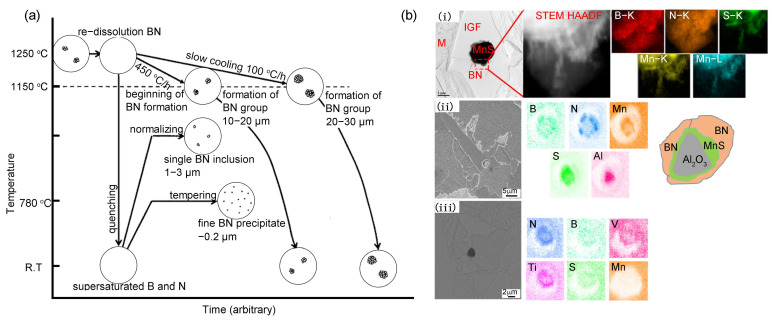
(**a**) Schematic diagram of BN formation behaviors in P122 steel under various heat treatments [69]. (**b**) The SEM and HADDF image of IGF at a 700 °C (**i**) and CGHAZ (**ii**,**iii**) [70].

The addition of trace concentrations of B in steel can significantly enhances its hardenability, which is widely utilized in the manufacture of high-strength, extra-thick plates featuring martensitic or bainitic microstructures [64,65]. Generally, the segregation of B atoms along austenitic grain boundaries reduces grain boundary energy, thereby hindering the nucleation of ferrite and bainite phases [66]. Chen et al. [33,67] delved into the impact of B content on the CGHAZ of G115 martensitic heat-resistant steel during welding and found that B segregation at austenitic grain boundaries promotes the formation of high-melting-point carbides, which impede austenite nucleation and growth, delay the austenitizing process, and reinforce carbide thermal stability, thus enhancing microhardness in the CGHAZ. Liu et al. [68] found that by elevating the B content from 0.0002 wt% to 0.0024 wt% in EH40 shipbuilding steel plates at a heat input of 400 kJ cm⁻¹, the segregation of B at grain boundaries effectively mitigated the size of grain boundary ferrite and promoted the formation of acicular ferrite, thereby enhancing the impact toughness of the CGHAZ. In low-alloy steels, B can form Fe_23_(B,C)_6_ or BN inclusions, which modulate the toughness of the HAZ. Sakuraya et al. [69] examined the effect of B on P122 heat-resistant steel and discovered that BN precipitation initiates during the cooling process after hot forging or rolling, within the 1150 °C to 1200 °C range, when B and N concentrations are 0.003% and 0.06%, respectively. The precipitate particle size diminishes with accelerated cooling rates but fails to precipitate at extremely rapid rates, similar to water quenching (Figure 6a). Shi et al. [70] investigated the influence of B on the intragranular ferrite nucleation mechanism in the CGHAZ of low-carbon V-N-Ti steel and demonstrated that the addition of B promotes the development of PF, which acts as a nucleation site for intragranular ferrite (IGF). Notably, when B is combined with complex precipitates, including (Ti,V)(C,N)-MnS, MnS, and Al_2_O_3_-MnS, there will form the cap structure containing a higher fraction of B and N comparing with other regions (Figure 6b(i)). From the analysis of high-angle annular dark field scanning transmission electron microscopy (HAADF STEM) with the observed precipitate morphology, it was determined that the cap primarily consisted of BN (Figure 6b(i)), which was further verified by the scanning electron microscope (SEM) images and corresponding energy dispersive spectrometer (EDS) analysis of the CGHAZ (Figure 6b(ii,iii)). The formed BN-capped structure can significantly enhance IGF nucleation within the CGHAZ, thus optimizing the toughness and low-temperature properties of steels. Yamamoto et al. [71] further elucidated that the addition of B to steel containing Ti_2_O_3_ promotes preferential nucleation of MnS and TiN due to the presence of cation vacancies in Ti_2_O_3_. This phenomenon is enhanced by B diffusion, which averts B segregation at interfaces. Consequently, the acceleration of fine intragranular ferrite formation occurs, refining the microstructure of HAZ and ultimately improving the toughness of CGHAZ. However, Melloy et al. [72] and Hatano et al. [35] cautioned that excessive B addition, i.e., exceeding 30 ppm, will diminish the toughness of the HAZ with increasing B content.

### 2.6. V

**Figure 7:**
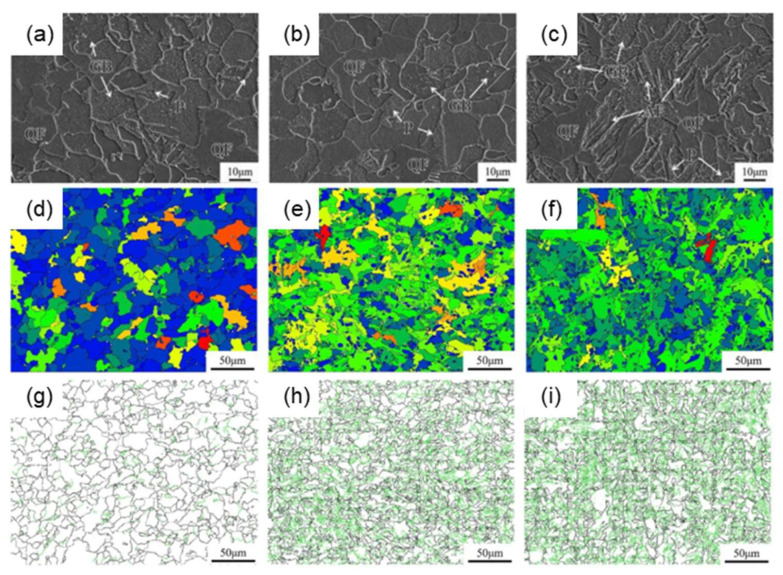
(**a**–**c**) SEM of HAZ after performing the welding process on the specimens: (**a**) EH36 steel, (**b**) EH36 steel with Mg treatment and (**c**) EH36 steel with Mg treatment; (**d**–**f**) Effective grain size of HAZ after performing the welding process on the specimens: (**d**) EH36 steel, (**e**) EH36 steel with Mg treatment, and (**f**) EH36 steel with Mg treatment. (**g**–**i**) The distribution of grain boundary orientation of HAZ after performing the welding process on the specimens: (**g**) EH36 steel, (**h**) EH36 steel with Mg treatment. and (**i**) EH36 steel with Mg treatment. In figures (**g**,**h**), the black line represents the small-angle grain boundary, which ranges from 2° to 15°, while the green line signifies the large-angle grain boundary, exceeding 15° [75].

The incorporation of V into high-nitrogen steel enhances its strength through two primary mechanisms: grain refinement and precipitation strengthening [73]. Vervynckt et al. [32] observed that V exhibits limited precipitation during the austenitic phase, enabling it to contribute effectively to precipitation strengthening either during or after the *γ* to *α* transformation. In this way, the strengthening effect of V(C,N) precipitation in ferrite significantly contributes to the overall enhancement of steel strength. Additionally, V exerts a grain -refining influence HSLA steel [74], and the presence of V(C,N) inclusions fosters intragranular nucleation of acicular ferrite, thereby enhancing the toughness of the CGHAZ. Bian et al. [75] investigated the welding of the HAZ in EH36 steel and observed that the integration of Mg deoxidation treatment with 0.028% of V effectively diminishes the quantity and dimensions of inclusions, while concurrently mitigating the austenite grain size at elevated temperatures, thus elevating the nucleation temperature of AF. Under a heat input of 100 kJ cm^−1^, the microstructure of EH36 steel and that treated solely with Mg primarily comprises PF, pearlite, and granular bainite. In contrast, the microstructure of EH36 steel treated with both Mg and V exhibits the presence of AF, in addition to PF, pearlite, and granular bainite (Figure 7a–c). Furthermore, after adding V, the effective average grain sizes were decreased from 8.3 to 5.7 μm (Figure 7d–f), and the proportion of large-angle grain boundaries obviously increases (Figure 7g–i), which may be attributable to the joint effect of austenite grain refinement and the AF nucleation promoted by the particles of vanadium nitride.

Hu et al. [76] also found that a small lattice mismatch between ferrite and vanadium nitride facilitated the formation of ultra-fine-grained ferrite during the welding process with the nucleation of V(C,N) precipitates in low-carbon V-N steel, which refine the M/A constituent and reduce the hardness by consuming carbon content, leading to an improvement in toughness of CGHAZ. Miyamoto et al. [77] examined the intragranular ferrite nucleation mechanism involving V(CN) + MnS particles and observed that V(CN) particles precipitating on MnS surfaces exhibit a favorable Baker–Nutting orientation relationship with α-ferrites. This orientation reduces interfacial energy during nucleation, fostering the formation of AF, with the V(CN)/austenite interphase boundary serving as a preferred nucleation site. Similarly, Shim et al.’s [78] investigation into the nucleation of intragranular ferrite with Al_2_O_3_ + VN particles reported analogous findings to the aforementioned literature. Zou et al. [79] conducted a comprehensive study on the impact of inclusions on the microstructural characteristics of the CGHAZ in Al–Ti–V–N steel. Their observations revealed the coexistence of a small proportion of AF and a substantial amount of PF within the microstructure. This unique microstructural composition was attributed to the pivotal role of micrometer-sized inclusions, including MnS, TiN, V(CN), and CuS, which facilitated the nucleation of both AF and PF. The mechanism of nucleation of AF and PF induced by micron-sized inclusions can be summarized as follows: 1. Role of MnS in forming Mn-depletion zone (MDZ): MnS inclusions create a MDZ with a thickness ranging from 0.35 to 3.5 μm around them. This MDZ enhances the nucleation driving force, thereby facilitating the formation of both AF and PF. Additionally, the MDZ surrounding the MnS contributes to the formation of AF on the upper left side of the inclusion. 2. Orientation relationships reducing interfacial energy: On the one hand, TiN exhibits a B-N orientation relationship with α-Fe, which effectively decreases the interfacial energy of α-Fe nucleation. This relationship is conducive to the nucleation of both AF and PF. On the other hand, V(CN) maintains a B-N or nearly B-N orientation relationship with α-Fe, which also reduces the nucleation interfacial energy. This leads to the preferential nucleation of α-Fe on V(CN) and promotes the formation of AF. PF. 3. Misfit Reduction by CuS: The misfit between CuS and ferrite is significantly smaller than that between CuS and austenite. This substantial reduction in nucleation interfacial energy may play a crucial role in the nucleation of AF and PF around inclusions containing CuS. For *t*_8/5_ of 400 and 500 s at heat inputs greater than 400 kJ cm^−1^, the average impact energy of Al-Ti-V steel reached 283 and 333 J at −20 °C, respectively.

### 2.7. N

**Figure 8:**
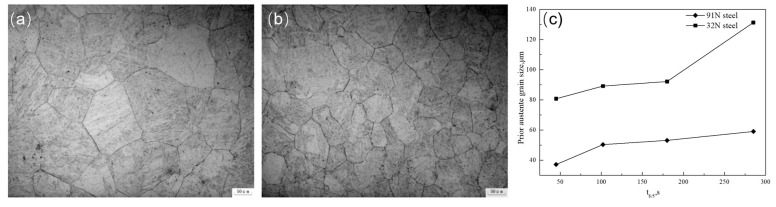
Prior austenite grain size of experiment steels quenched at different cooling times of *t*_8/5_ 180 s: (**a**) V–Ti steel, (**b**) V–Ti steel observed by OM, (**c**) sized against cooling time *t*_8/5_ from 45 s to 285 s [82].

The addition of N can fortify the strength and elevate the thermal stability of austenite, thus enhancing the strengthening of HSLA steel [80]. In comparison to carbon elements, N exhibits a diminished size, leading to the formation of smaller nitrides with lower surface energy. These nitride nanoparticles serve as effective anchors for the pre-existing austenite grains within the CGHAZ, simultaneously enhancing the steel’s strength through a grain refinement mechanism [81]. Shi et al. [82] systematically studied the effect of N on the microstructural evolution and mechanical properties of simulated CGHAZ in V-Ti and V-N-Ti steels and observed that, compared to V-Ti steel, the prior austenite grain size in V-N-Ti steel was notably smaller across various cooling time (*t*_8/5_) conditions (Figure 8).

**Figure 9:**
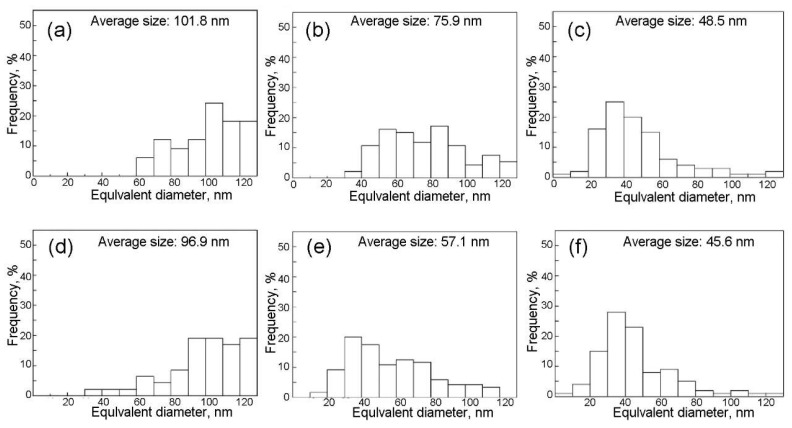
Size distribution of precipitates in the base plate and simulated CGHAZ. Steel with 0.0044% N: (**a**,**b**); Steel with 0.0094% N: (**c**,**d**); Steel with 0.0190% N: (**e**,**f**). Base plate: (**a**,**c**,**e**); Simulated CGHAZ: (**b**,**d**,**f**) [83].

Based on the analysis of particle size distributions, Shi et al. also [83] discovered that the base plate and CGHAZ exhibited a notably higher proportion of fine particles with the N content increase from 0.0044 wt% to 0.0094 wt% (Figure 9), which indicates the inhibitory effect of N addition on particle coarsening, resulting in the achievement of finer particles. In addition, the addition of N significantly facilitated the transformation of the ferrite structure within the simulated CGHAZ. As the nitrogen content increased to about 0.015wt%, the primary microstructure of the CGHAZ transitioned from grain boundary ferrite (GBF) to intergranular acicular ferrite (IGAF) and PF [82,83,84,85]. As the nitrogen content rose, both the number of fine and coarse particles increased. Fine precipitates effectively pinned the prior austenite grain boundaries (PAG), leading to a reduction in the PAG diameter.

Although the augmented N content enhanced the stability of austenite [86,87], it also significantly contributes to the homogenization of the distribution of M/A constituents and the refinement of M/A [84,88,89], transforming the internal substructure of M/A components from twin type to lath type, thereby altering the initiation and propagation mechanisms of cracks. As reported, the mechanism of improving both mechanical properties and toughness through the addition of nitrogen is attributed to its ability to facilitate the formation of (Ti,V) (CN) or V(C,N) precipitates [84,85]. These precipitates anchored the original austenite grain boundaries in the CGHAZ, fostering intragranular ferrite nucleation and preserving a distinct Baker–Nutting orientation relationship with the intragranular ferrite. This phenomenon further contributed to the refinement of the effective grain size and the enhancement of cleavage fracture stress in the simulated CGHAZ. Importantly, the refinement of both austenite grains and effective grain size effectively alleviated the detrimental effects of free nitrogen on the toughness of CGHAZ.

### 2.8. Ti

**Figure 10:**
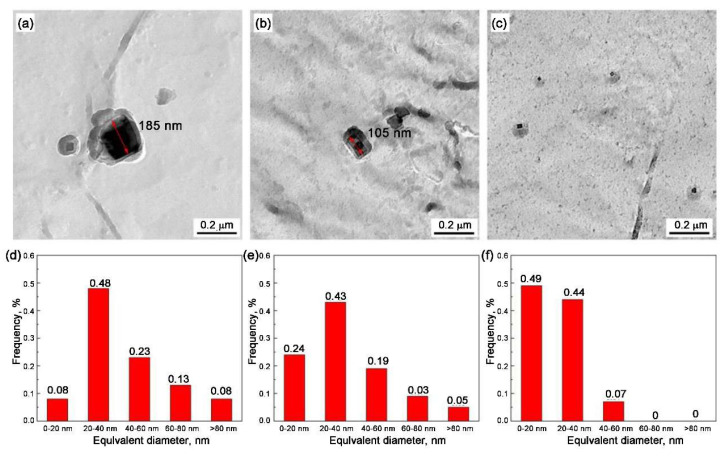
Size distributions of precipitates in high–heat–input welding steels with different Ti/N ratios: (**a**,**d**) Ti/N = 5.85; (**b**,**e**) Ti/N = 4.22; and (**c**,**f**) Ti/N = 2.82 [15].

Ti, a crucial micro-alloying constituent, is consistently employed as a stabilizing agent that interacts with carbon or nitrogen to generate precipitates. These precipitates, endowed with remarkable high-temperature stability, effectively hinder the coarsening of austenite grains under at high temperatures as well as stimulate IF nucleation, thereby enhancing the mechanical properties around the welded region in HSLA [90,91,92,93,94,95]. In the context of welding thermal cycling, TiN exhibits a pivotal role in suppressing austenite grain growth via a pronounced grain boundary pinning mechanism, which leads to a refinement of the microstructure, ultimately enhancing both the microstructural characteristics and the mechanical properties within the simulated CGHAZ [96,97,98]. When TiN particles are finely dispersed within the austenite matrix, they effectively hinder the migration of austenite grain boundaries, thus inhibiting grain growth. This inhibition is achieved by carefully controlling the total content and ratio of Ti and N to facilitate the precipitation and dispersion of TiN particles within the matrix under optimal conditions. However, excessively high concentrations of Ti and N can lead to premature precipitation and coarsening of TiN particles before solidification, diminishing their ability to pin austenite grain growth. Consequently, precise control of Ti and N content, along with the heat treatment process, is essential for optimizing the pinning effect of nanoscale TiN particles on austenite grain growth. Therefore, the Ti/N ratio exerts a particularly notable influence on the microstructure and properties of CGHAZ. Yan et al. [99] discovered that the presence of large TiN will induce poor low-temperature toughness and the enhancement of ductile-to-brittle transition temperature of steels, which can be addressed by control the ratio of Ti and N. Specifically, the Ti/N ratio should be controlled below the stoichiometric ratio of 3.42 to facilitate the precipitation of fine TiN particles, which can effectively impede the growth of austenite grains and slow down the coarsening rate of TiN, leading to a higher volume fraction of finer austenite grains [100]. Liu et al. [15] and Zhang et al. [101] demonstrated that in steel welding processes involving high-heat-input, a reduction of the Ti/N ratio to approximately 3 results in a refinement and more uniform distribution of TiN particles with less than 40 nm (Figure 10). This modification promotes an increase in the area fraction of IAF, thereby enhancing the proportion of ductile microstructures and the content of high-angle grain boundaries. Additionally, it mitigates the formation of locally high strain structures. Consequently, under heat inputs of 150 kJ cm^−1^ and 400 kJ cm^−1^, the Charpy impact-absorbed energy can attain values as high as 333 J and 183.3 J, respectively.

To attain a dispersed arrangement of fine-sized inclusions, the incorporation of strong deoxidants like Mg or Ca, in combination with Ti, is a commonly adopted approach. Song et al. [102] conducted a comparative study to evaluate the influence of Ti-Mg and Ti on the impact toughness of HAZ in steel and found that Ti-Mg treatment notably enhanced the AF content in the steel and diminished the initial austenitic grain size. Notably, during welding, the second-phase particles in the Ti-Mg-treated steel demonstrated a pronounced pinning effect and resistance to coarsening, facilitating grain refinement. Wang et al. [103] revealed that the oxide particles in Ti-Ca deoxidized steel were predominantly composed of the Ti-CaO-Mn-S complex, exhibiting a remarkably uniform distribution. These particles significantly facilitated IAF nucleation and augmented the intragranular bainitic structure, ultimately resulting in a notable enhancement in both strength and toughness. Additionally, the impact of Ti addition on V-N micro-alloyed steel was also investigated. Fang et al. [104] indicated that the introduction of Ti in V-N steel promoted the formation of TiN and facilitated the absorption of nitrogen from the matrix, thereby creating additional sites for the precipitation of V(C,N). As a result, a substantial number of circular precipitate particles were observed to surround the TiN in the titanium-containing steel.

## 3. Conclusions and Perspectives

This comprehensive review examines the intricate relationships between micro-alloying elements in high-strength, low-alloy (HSLA) steels during high-heat-input welding. The primary focus is on the differential effects of these elements on the heat-affected zone (HAZ) toughness. Elements such as cerium (Ce), magnesium (Mg), titanium (Ti), vanadium (V), nitrogen (N), and boron (B) exhibit positive impacts on HAZ toughness through various mechanisms. They facilitate the intragranular nucleation of acicular ferrite and hinder austenite grain growth. For instance, Ce modifies inclusions and anchors austenite grain boundaries, while also refining carbonitrides, thereby enhancing the mechanical properties of both the HAZ and the base metal. Mg, in conjunction with other elements, forms fine composite inclusions that impede austenite grain expansion and promote acicular ferrite nucleation. Ti encourages acicular ferrite nucleation within the HAZ, and by managing the Ti/N ratio and employing deoxidizers, the steel’s low-temperature toughness can be augmented. Additionally, B, in combination with V and N, influences phase nucleation, precipitation, and austenite grain growth, ultimately improving hardness and HAZ impact toughness.

Conversely, the effects of aluminum (Al) and niobium (Nb) on HAZ toughness are more complex and often detrimental. The influence of Al on coarse-grained heat-affected zone (CGHAZ) toughness remains controversial. Some studies suggest that high Al content can lead to increased lattice mismatch, inclusion count, and brittle microstructures, reducing impact toughness. However, other research indicates that Al may enhance toughness by decreasing the volume fraction of martensite-austenite constituents. On the other hand, Nb segregates at the inclusion–matrix interface, impeding acicular ferrite nucleation and promoting the formation of coarse bainitic ferrite, thereby degrading HAZ toughness.

In oxide metallurgy, the combination of these micro-alloying elements is pivotal. Elements such as Ti, Mg, Ce, B, V, and N work synergistically to promote acicular ferrite nucleation and inhibit austenite grain growth, thereby enhancing HAZ toughness. The selection of appropriate oxide metallurgy techniques and micro-alloying element combinations is crucial and should be tailored to specific application requirements.

Although tremendous achievements have been realized in both fundamental understanding and technological improvements, there are certain issues and problems that need to be addressed to improve the weldability of HSLA:

(1) Synergistic mechanism of microalloying elements

Understanding the interaction mechanisms between various micro-alloying elements is imperative. In-depth research should focus on how these elements collectively influence steel welding and mechanical properties. By exploring various combinations of micro-alloying elements, we can optimize oxide metallurgy effects. For example, studying the interplay between different oxide-forming processes and elements such as Ti, Nb, and V can help tailor steel properties for specific applications. This research may uncover novel methods to enhance the welding and mechanical properties of steel, potentially leading to the development of innovative alloy systems.

(2) Development of high-performance welding materials

As industries demand higher-performance HSLA steels, there is an urgent need to develop innovative oxide metallurgy techniques and micro-alloying element combinations that meet the stringent welding performance standards of modern applications. Investigating methods for achieving high-quality welding at low temperatures without preheating is particularly crucial. This would not only improve welding efficiency but also contribute to more sustainable manufacturing practices. Research could focus on understanding the fundamental principles of inclusion formation and microstructure evolution during welding to develop materials with superior performance.

(3) Combination of physical models and numerical simulations

Physical models and numerical simulations provide a powerful means to study the complex phenomena in HSLA steels during welding. These tools can be utilized to examine the interaction between rare earth elements such as Ce and other micro-alloying elements. For instance, simulations can help elucidate how Ce affects inclusion formation and microstructural dynamics in the HAZ. Numerical models can also predict the mechanical properties and toughness of steel under various welding conditions. This information can guide experimental work, reducing the time and cost associated with trial-and-error methods. Furthermore, it can provide a theoretical foundation for optimizing welding processes and alloy compositions.

(4) Effect of inclusions on AF nucleation mechanism

The specific impact of different inclusion types on the acicular ferrite (AF) nucleation mechanism is not fully comprehended. Further research is needed to clarify how the composition, size, and distribution of inclusions influence AF nucleation. Advanced microscopy techniques could be employed to study the interface between inclusions and the matrix at the atomic scale. By understanding these mechanisms, we can manipulate inclusion characteristics to optimize the steel microstructure. For example, precisely controlling the formation of certain inclusion types could enhance AF nucleation, leading to improved toughness and mechanical properties.

(5) Optimization of microstructure and properties of CGHAZ

The CGHAZ often exhibits inferior mechanical properties compared to the base metal. Future research should explore methods to optimize its microstructure and properties. This could involve adjusting welding parameters such as heat input, cooling rate, and welding speed. Additionally, modifying the steel composition by carefully selecting and adjusting the content of micro-alloying elements can have a significant impact. By understanding how these factors interact, we can develop strategies to refine the microstructure in the CGHAZ, increase the proportion of beneficial phases like acicular ferrite, and ultimately enhance the overall mechanical properties and toughness of the steel.

## Data Availability

No new data were created or analyzed in this study.

## Abbreviations

The following abbreviations are used in this manuscript:

HSLA	High-strength low-alloy
CGHAZ	Coarse-grained heat-affected zone
HAZ	Heat-affected zone
FGHAZ	Fine-grained or recrystallization heat-affected zone
ICHAZ	Intercritical heat-affected zone
M/A islands	Martensite-austenite (M/A) islands
AF	Acicular ferrite
Ce	Cerium
Al	Aluminum
Mg	Magnesium
PF	Polygonal ferrite
IAF	Intragranular acicular ferrite
A	Al deoxidation
LM	Low-content Mg deoxidation
HM	High-content Mg deoxidation
Nb	Niobium
B	Boron
IGF	Intragranular ferrite
HAADF STEM	High-angle annular dark field scanning transmission electron microscopy
SEM	Scanning electron microscope
EDS	Energy dispersive spectrometer
V	Vanadium
MDZ	Mn-depletion zone
N	Nitrogen
GBF	Grain boundary ferrite
IGAF	Intergranular acicular ferrite
PAG	Prior austenite grain boundaries
Ti	Titanium
